# Efficacy of amniotic membrane with coronally advanced flap in the treatment of gingival recession: an updated systematic review and meta-analysis

**DOI:** 10.1186/s12903-023-03825-y

**Published:** 2024-01-25

**Authors:** Reham Abdel-Fatah, Wafaa Saleh

**Affiliations:** Oral Medicine, Periodontology, Diagnosis and Oral Radiology Department, Faculty of Dentistry, Mansoura University, Mansoura, Egypt

**Keywords:** Amniotic membrane, Coronally advanced flap, Gingival recession, Systematic review, Meta-analysis

## Abstract

**Objectives:**

This systematic review aims to evaluate the efficacy of combining the amniotic membrane (AM) with the coronally advanced flap (CAF) in the treatment of Miller class I and II gingival recession (GR).

**Methods:**

The protocol of this updated PRISMA-compliant systematic review was registered in PROSPERO (CRD42023431501). The following treatment outcomes were recorded; recession depth (RD), recession width (RW), width of keratinized gingiva (WKG), and clinical attachment level (CAL). We searched the following databases: MEDLINE, Cochrane Library, Google Scholar, EMBASE, Web of Science, and Science Direct.

**Results:**

Two independent reviewers screened the selected articles. Twenty-two eligible articles were extracted, with 689 sites of GR in 481 patients. No statistically significant difference was found in RD, RW, WKG, and CAL between (AM&CAF) in comparison to control groups. However, the subgroup analysis showed statistically significant differences in RD between the (AM & CAF) group v/s (CAF) alone (P = 0.004). Moreover, the subgroup analysis of the WKG showed statistically significant differences between (AM & CAF) v/s (CAF&CM) (p = 0.04). Additionally, a statistically significant difference was found in the subgroup analysis of CAL between both (AM & CAF) group v/s (CAF) alone (p = 0.0009).

**Conclusion:**

With the limitations of this meta-analysis due to short follow-up periods (6 months), the AM can be considered a viable treatment option for GR defects with satisfactory treatment outcomes comparable to other previously investigated treatment modalities.

**Clinical significance:**

While AM showed various beneficial properties as an ideal membrane for the coverage of GR, future studies are required to completely understand the potential application of AM in the treatment of GR.

**Supplementary Information:**

The online version contains supplementary material available at 10.1186/s12903-023-03825-y.

## Introduction

Gingival recession(GR) is a prevalent dental condition in which the gingival margin migrates apically to the cementoenamel junction resulting in root exposure, hypersensitivity, and the unsightly appearance of the teeth [1]. Moreover, the incidence of GR is approximately 54% in young adults aged 26–35 years [2]. There are several causes of GR, including plaque and calculus accumulation, periodontal diseases, traumatic tooth brushing, malocclusion, orthodontic treatment, genetic factors, and anatomical factors. Diagnosis of GR can be performed through clinical and radiographic examinations. The severity of GR can be assessed through clinical evaluation by measuring the amount of recession, the thickness of keratinized gingiva, the pocket depth (PD), and the amount of attachment loss [3, 4].

Treatment of GR receives significant attention from patients due to aesthetic and functional purposes. It may indicate a more serious underlying dental problem that requires treatment to prevent further damage. Several treatment modalities are available for GR depending on the underlying cause and the severity of the condition.Treatment options range from improving oral hygiene to surgical intervention procedures. The main goals of the treatment are to cover the exposed root surface, prevent further damage, and enhance the esthetic appearance of the gingiva and teeth [5].

Surgical interventions may be necessary for more severe cases of GR. Different flap techniques have been utilized with different root surface bio-modifications. The gold standard treatment of choice is the coronally advanced flap(CAF) combined with the sub-epithelial connective tissue graft (SCTG) [6, 7]. Several types of resorbable and non-resorbable membranes have been used as a substitute for connective tissue graft(CTG) in guided tissue regeneration techniques [8]. Resorbable membranes including collagen, synthetic, and recently used Amniotic membranes (AMs) are preferred to non-resorbable ones regarding the elimination of the second intervention for membrane removal [9].

Recently, the AM has gained popularity in medicine due to its various applications in eye surgeries, orthopedics, gynecologic surgeries, burns, as well as biological dressings for wounds. It was recommended because of postoperative pain reduction, damaged organ reconstruction, and tissue adhesion prevention [10].

AM refers to the innermost placental layer that lines the amniotic cavity. It consists of an epithelial cell layer, basement membrane, and connective tissue which is non-vascular. Various adhesion molecules were detected in the basement membrane including collagen Types III, IV, and V in addition to laminins and fibronectin [11, 12]. In Addition, various stem cells and growth factors were extracted from AM. Moreover, the AM can provide neovascularization, early physiologic granulation tissue formation, and reduce the host response due to the prevention of migration of polymorphonuclear cells [13].

Numerous studies have unequivocally demonstrated that AM can be employed as a viable alternative to CTGs in the realm of guided tissue regeneration techniques. This application effectively facilitates the augmentation of gingival thickness (GT) and the comprehensive coverage of recessed areas, leading to a substantial enhancement in aesthetic outcomes. Despite the considerable promise of AM in GR treatment, there is a pressing need for additional research to comprehensively assess its long-term efficacy and safety profile [14–18].

Thus, we conducted the current updated systematic review and meta-analysis to evaluate the effectiveness of AM combined with CAF in comparison with different biomaterials utilized for GR coverage.

## Materials and methods

### Protocol and registration

The current systematic review was executed following the guidelines of the Preferred Reporting Items of Systematic Reviews and Meta-Analysis (PRISMA). It was duly registered in the PROSPERO database under the registration number (CRD42023431501). The study protocol was designed following the Cochrane Handbook for Systematic Reviews of Interventions [19].

### Focused PICOS questions

The following PICOS model was employed for this review:

**P**— Patients with localized GR Millers Class I or Class II.

**I**— Intervention being evaluated was the CAF surgical technique used with AM.

**C**— Comparison was done with CAF surgical technique alone or in combination with different biomaterials other than AM.

**O**— Outcome measures the primary outcome measures encompassed changes in recession depth (RD), recession width (RW), width of keratinized gingiva (WKG), and the percentage of root coverage. Meanwhile, the secondary outcome measures included shifts in the clinical attachment level (CAL) and probing pocket depth (PPD).

**S**— Studies the included studies were restricted to studies applied to human GR defects that were published only in the English language.

### Search strategy

A thorough electronic database search was conducted, extending up to July 2023. All studies pertaining to human gingival recession (GR) that employed amniotic membrane (AM) and were published in the English language were meticulously curated from the following databases: MEDLINE (PubMed), Cochrane Library, Google Scholar, EMBASE, Web of Science, and Science Direct.

The electronic search encompassed the following key terms: (“Amniotic membrane” OR “placental membrane”) AND (“Gingival recessions” OR “localized gingival recession” OR “Miller class I and II gingival recession”).

### Inclusion criteria

We included systematically healthy individuals in the age range (18–55 years old) with localized GR defects without interproximal tissue loss (Miller Class I or II).

Our inclusion criteria were designed to select studies that met specific predefined criteria:

- Studies published in English.
- Randomized clinical trials (RCTs) and observational studies.
- Studies evaluating the use of AM with CAF in the treatment of GR.
- Studies that reported primary and secondary clinical outcomes of interest.

### Exclusion criteria

This systematic review excluded the case series, case reports, and the studies conducted on systemically compromised patients, pregnant, lactating mothers, patients with a history of periodontal surgery in the last six months and cases with fenestration and dehiscence.

### Article selection process

**Fig. 1:**
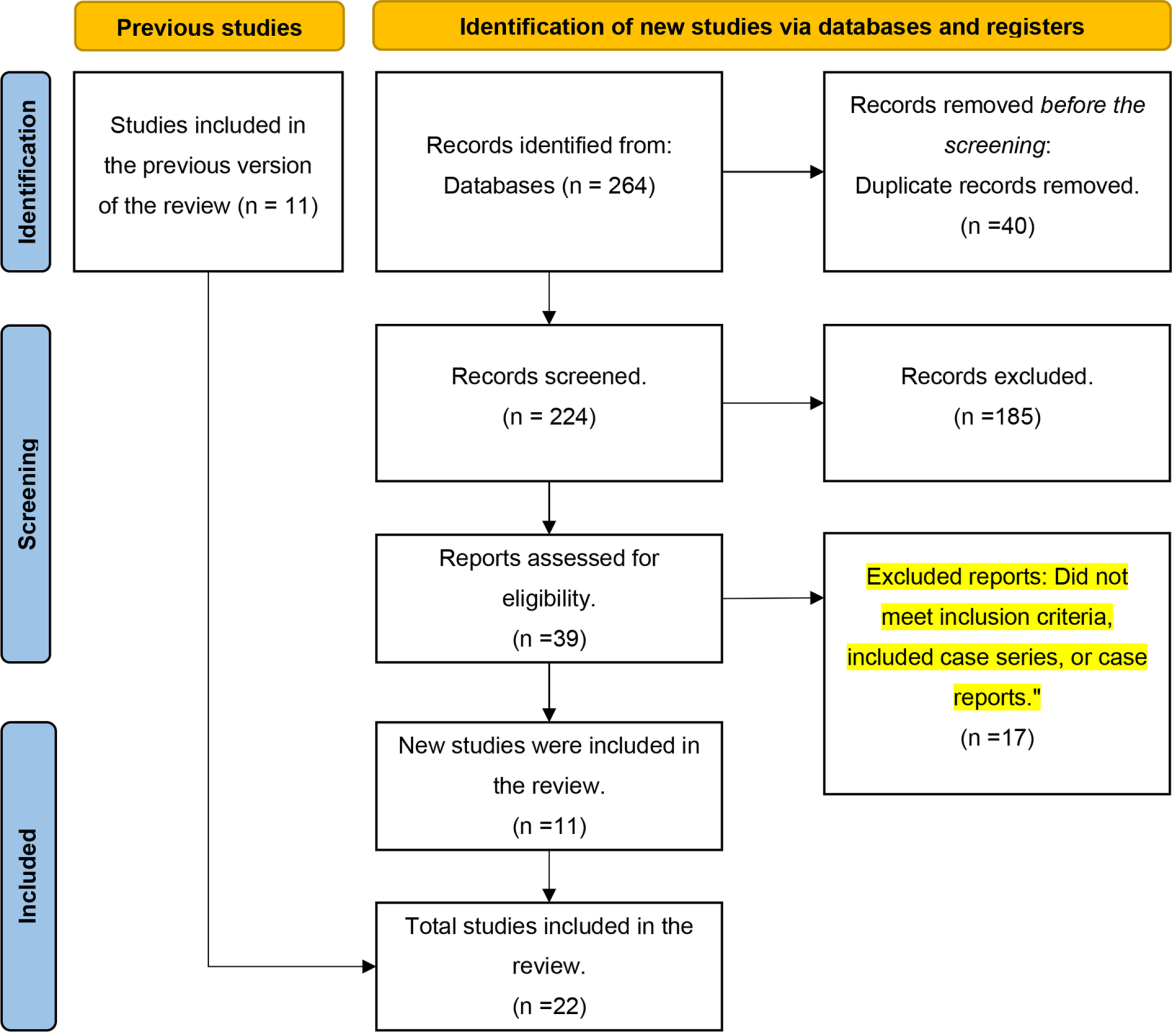
PRISMA flow diagram for updated systematic review which included searches of databases and registers only

The initial screening involved two independent reviewers, R.A and W.S, for the selection of eligible articles. Subsequently, the full texts of the chosen articles underwent scrutiny, encompassing the removal of any duplications, ultimately leading to a consensus-based final selection by both reviewers. Any discrepancies between the two reviewers were amicably resolved through open discussion. Notably, case reports and case series were intentionally excluded from the ongoing systematic review. Studies failing to align with the previously specified inclusion criteria (as depicted in Fig. 1) were also excluded from the analysis.

The data of included studies was then extracted in a Microsoft Excel sheet (Tables 1, 2 and 3). Any disagreement between the investigators was resolved by discussion.

**Table 1:** Study design of included studies

Author and Year	Study Design	Randomization	Surgical technique	
			Test (group I)	Control (group II)
[20]	Randomized controlled clinical study	Performed	CAF + AM	CAF + PRF
[21]	A Clinical Study	Performed	CAF + AM	CAF + Gengigel
[22]	Randomized, parallel-mouth controlled study	Performed	CAF + AM	CAF + PRF
[16]	Clinical study (split mouth)	Performed	CAF + AM	CAF + CM
[23]	Clinical study	Performed	CAF + AM Demineralized freeze-dried bone allograft (DFDBA)	CAF + CM + DFDBA
[24]	A randomized controlled study. (Splitmouth study)	Performed	CAF + AM	CAF + functionally graded membrane (FGM)
[25]	Randomized controlled clinical trial (Split mouth)	Performed	CAF + AM	CAF only
[15]	Randomized controlled clinical trial (split-mouth)	Performed	CAF + AM	CAF + PRF
[26]	Randomized study (Spilt mouth)	Performed	CAF + AM	CAF alone
[14]	Randomized controlled study	Performed	CAF + AM	CAF + CTG
[27]	randomized controlled trial (split mouth)	Performed	CAF + AM	CAF + CM
[28]	clinical study (split mouth)	Performed	CAF + AM	CAF only
[29]	Clinical study	Performed	CAF + AM	CAF + PRF
[30]	Randomized controlled clinical trial. (Split–mouth)	Performed	CAF + AM	CAF only
[31]	randomized clinical trial(split-mouth)	Performed	CAF + AM	CAF + SCTG
[32]	Clinical study	Performed	CAF + AM	CAF + PRF
[33]	Clinical study (Split mouth)	Performed	CAF + AM	CAF + collagen membrane
[34]	Randomized controlled clinical trial	Performed	CAF + AM (Microsurgical technique)	CAF + AM (Macro-surgical technique)
[17]	Clinical study (split mouth)	performed	CAF + AM	CAF only
[35]	clinical study	Performed	CAF + AM	CAF only
[18]	Randomized controlled clinical trial	Performed	CAF + AM	CAF + PRF
[36]	Clinical study	Performed	CAF + AM	CAF + PRF

PRF, platelet rich fibrin; SCTG, sub-epithelial connective tissue graft; CM, chorion membrane

**Table 2:** General characteristics of included studies

Author & Year	Recession Type	Recession location	No. of Patients	Smokers	No. of defects (Test/Control)	Age Range	Male/Female	Follow-up (Months)	Percentage of Root Coverage (%)		Authors conclusion
									Test	Control	
[20]	Miller’s Class I or Class II GR	Maxillary anterior teeth region	23	excluded	45	> 18 y	M;18 F;5	3,6	36%	56%	It showed better root coverage when PRF or AM were used in conjunction with CAF as compared to CAF alone.
[21]	Miller’s class I and class II GR	Maxillary anterior teeth region	45	excluded	45	21–53 y	M:36 F:9	6	NR	NR	CAF used with AM showed favorable results in the treatment of Miller’s class I and II GR.
[22]	Miller’s Class I GR	Maxillary and nine mandibular defects	10	excluded	20	NR	M:10	6,18	NR	NR	AM demonstrated a higher percentage of root coverage than PRF when both were combined with CAF.
[16]	Single bilateral Miller’s Class I or Class II GR	NR	12	excluded	24	28-40y	NR	1,3,6	22%	28%	CM showed more root coverage with a reduction in recession depth while AM showed more CAL gain.
[23]	Miller’s Class I and II recession defects	Maxillary anterior and premolars	30	excluded	30	20–50	NR	3	NR	NR	CAF combined with AM and DFDBA showed better results compared to CAF alone in Miller’s Class I and II GRs.
[24]	buccal/labial vertical GR defects more than or equal to 2 mm.	NR	9	Excluded	22	30–55 y	M:8 F:1	3,6	NR	NR	Both FGM and AM showed the same regenerative potential.
[25]	Miller’s class I and class II	NR	15	excluded	30	NR	NR	1, 3,6	NR	NR	CAF alone showed better results regarding recession reduction in Miller’s class I & II GRs than CAF with AM.
[15]	Bilateral Miller class I GR	Maxillary and mandibular canines	15	Excluded	30	21–52 y	M:5 F:10	6	76.47%	56.94%	CAF with both PRF and AM can be successfully used to treat class I GR with AM gives better outcomes.
[26]	bilateral Miller’s class I and II GR	Maxillary and mandibular anterior and premolar regions	15	excluded	60	23–55 y	M:11 F:4	3,6	NR	NR	AM with the CAF showed reliable root coverage with favorable healing outcomes as compared to CAF alone.
[14]	Miller’s class I and II GR	Maxillary and mandibular anterior and premolar regions	22	excluded	71	> 18 y	NR	3,6	67%	54%	AM may substitute CTG in root coverage procedures and RD reduction.
[27]	Miller class I and II	NR	10	excluded	20	20–50 y	NR	6	NR	NR	Both AM and CM can be safely used in the treatment of GR defects and to augment the gingival phenotype.
[28]	Bilateral Miller’s class I GR defect	NR	10	excluded	20	18–40 y	M:5 F:5	3,6	NR	NR	CAF with AM can be used for treating Miller’s Class I GR defects.
[29]	Miller’s class I and II GR	NR	30	Excluded	30	18–55 y	M:15 F:15	3,6	NR	NR	Both AM and PRF were equally effective in terms of recession coverage and increased WKG.
[30]	Isolated bilateral Miller’s class I GR defects	Maxillary and mandibular anterior and premolar regions	51	Excluded	102	18–40 y	NR	6	85%	81%	CAF with AM proved to be fruitful in comparison with CAF alone.
[31]	Miller class I and II buccal recessions	Maxillary and mandibular anterior and premolar regions	11	excluded	30	34 ± 12 y	NR	1,3,6	63.18%	75.54%	AM with CAF may be relatively comparable with gold standard SCTG with CAF for the treatment of Miller class I and II GR.
[32]	Single Miller’s Class I or Class II GR	NR	24	Excluded	24	20–60 y	NR	3,6	77%	62%	AM showed more root coverage than PRF.
[33]	Isolated bilateral Miller’s Class I or Class II GR	NR	12	Excluded	24	18–40 y	F:7 M:5	3,6	73.31%	59.03%	Collagen membrane and AM are equally efficacious.
[34]	Single Miller’s Class I or Class II GR	Maxillary anterior, premolar, and molar regions	24	Excluded	24	22–41 y	F:4 M:20	3,6	NR	NR	AM with a microsurgical approach showed better root coverage outcomes and stable results at the end of the study period.
[17]	Single bilateral Miller’s Class I or Class II GR	NR	9	NR	18	NR	NR	3,6	NR	NR	The combination of CAF with AM provided additional outcomes in the treatment of GR.
[35]	bilaterally localized Miller’s class I or II GR	Maxillary and mandibular anterior region	5	excluded	10	30–40 y	NR	3	NR	NR	AM with CAF did not influence the clinical outcome of the root coverage procedure.
[18]	Single Miller’s Class I GR	Maxillary and mandibular sites	16	Excluded	20	20–45 y	M:10	6,18	NR	NR	AM showed better root coverage as compared to PRF.
[36]	Single Miller’s Class I or Class II GR	Maxillary anterior and premolars region	30	Excluded	30	18–55 y	NR	3,6,9	At 6 months 67.6% At 9 months 64.27%	At 6 months 65.27% At 9months 57.0%	AM was more effective in terms of increasing WKT.

**Table 3:** Clinical characteristics of included studies at 6 months follow up

Author and Year	Groups	Recession depth (mm)		Recession width(mm)		Width of keratinized gingiva (mm)		Probing depth(mm)		Clinical attachment level (mm)	
		Baseline	Final	Baseline	final	Baseline	Final	Baseline	Final	Baseline	Final
[20]	Group(I) Group (II)	1.87 ± 0.74 2.60 ± 0.83	1.20 ± 1.47 1.20 ± 1.21	4.20 ± 0.77 4.33 ± 0.62	2.73 ± 1.44 2.60 ± 1.99	3.67 ± 0.98 3.60 ± 1.12	4.60 ± 1.59 4.80 ± 1.66	1.93 ± 0.70 1.93 ± 1.10	1.67 ± 0.82 1.93 ± 0.80	3.67 ± 1.11 4.33 ± 1.63	2.47 ± 1.36 2.87 ± 1.88
[21]	Group(I) Group (II)	NR NR	0.87 ± 0.35 0.93 ± 0.26	NR NR	1.60 ± 0.63 1.33 ± 0.49	NR		NR	0.20 ± 0.41 0.33 ± 0.49	NR	1.07 ± 0.59 1.27 ± 0.59
[22]	Group(I) Group (II)	3 ± 0.84 2.5 ± 0.84	0.4 ± 1.713 0.7 ± 1.713	NR		1.5 ± 0.388 2 ± 0.388	2.3 ± 1.466 3 ± 1.466	NR		NR	
[16]	Group(I) Group (II)	7.33 ± 1.44 7.00 ± 1.86	5.75 ± 1.14 5.00 ± 1.54	9.00 ± 1.71 9.08 ± 1.78	6.50 ± 1.51 6.92 ± 1.51	3.42 ± 0.51 3.33 ± 0.49	4.42 ± 0.51 4.75 ± 0.45	NR NR	NR NR	9.00 + 1.86 9.331 + 1.44	6.83 + 1.53 7.75 + 1.22
[23]	Group(I) Group (II)	3.00 ± 0.667 3.20 ± 0.919	(at 3 months)1.10 ± 0.738 (at 3 months)0.70 ± 0.823	3.20 ± 0.789 2.40 ± 0.516	(at 3 months)1.40 ± 0.667 (at 3 months)0.50 ± 0.527	2.5 ± 0.632 2.82 ± 0.487	(at 3 months)3.53 ± 0.749 (at 3 months)3.6 ± 0.612	NR		11.10 ± 0.876 9.90 ± 3.28	(at 3 months)8.70 ± 1.252 (at 3 months)7.30 ± 2.452
[24]	Group(I) Group (II)	NR		NR		NR		NR		NR	
[25]	Group(I) Group (II)	2.53 ± 0.83 2.60 ± 0.83	1.90 ± 1.54 1.67 ± 1.18	NR		NR		NR		NR	
[15]	Group(I) Group (II)	2.17–0.61 2.10–0.58	0.12–0.21 0.23–0.27	NR		NR		NR		NR	
[26]	Group(I) Group (II)	2.87 ± 0.9 2.63 ± 0.765	1.00 ± 0.00 1.43 ± 0.568	2.13 ± 0.776 2.47 ± 0.681	1.00 ± 0.000 1.20 ± 0.484	1.50 ± 0.731 1.73 ± 0.785	3.80 ± 0.551 2.43 ± 0.971	1.90 ± 0.803 1.87 ± 1.042	1.20 ± 0.407 1.40 ± 0.498	4.93 ± 1.143 4.50 ± 1.408	2.20 ± 0.407 2.83 ± 0.791
[14]	Group(I) Group (II)	3.43 ± 1.741 4.12 ± 1.986	1.13 ± 1.452 1.88 ± 1.467	3.89 ± 1.192 4.38 ± 0.852	1.25 ± 0.496 2.93 ± 1.801	2.76 ± 1.664 2.39 ± 1.277	3.44 ± 1.298 3.34 ± 1.610	NR		4.99 ± 1.403 5.98 ± 2.055	2.64 ± 1.474 3.82 ± 1.593
[27]	Group(I) Group (II)	8.00 ± 1.56 7.90 ± 1.52	7.00 ± 1.69 7.10 ± 1.37	NR		NR		NR		9.60 ± 2.22 9.50 ± 1.50	8.50 ± 2.01 8.40 ± 1.34
[28]	Group(I) Group (II)	2.9 ± 0.87 2.5 ± 0.90	0.4 ± 0.51 0.3 ± 0.48	3.2 ± 0.42 3 ± 0.81	0.5 ± 0.52 0.40 ± 0.51	2.9 ± 0.73 3.0 ± 0.66	4.7 ± 0.67 4.3 ± 0.67	1.3 ± 0.48 1.2 ± 0.42	1.0 ± 0.0 1.1 ± 0.31	4.3 ± 1.15 3.6 ± 0.84	1.4 ± 0.51 1.3 ± 0.48
[29]	Group(I) Group (II)	2.800 ± 0.862 2.733 ± 0.799	1.000 ± 1.000 1.400 ± 0.633	NR NR		3.000 ± 0.535 2.733 ± 0.704	3.667 ± 0.488 3.267 ± 0.594	NR NR		NR NR	
[30]	Group(I) Group (II)	2.95 ± 0.89 2.70 ± 0.85	0.43 ± 0.45 0.50 ± 0.45	3.10 ± 0.41 3.20 ± 0.79	0.49 ± 0.50 0.55 ± 0.47	3.00 ± 0.75 3.10 ± 0.71	4.62 ± 0.22 4.62 ± 0.25	1.20 ± 0.49 1.20 ± 0.45	1.05 ± 0.12 1.13 ± 0.39	4.40 ± 1.16 4.10 ± 0.89	1.53 ± 0.52 1.64 ± 0.50
[31]	Group(I) Group (II)	3.13 ± 0.4 3.43 ± 0.63	1.13 ± 1.26 0.8 ± 0.8	4.33 ± 0.84 4.5 ± 0.5	1.66 ± 1.67 2.1 ± 1.04	3.13 ± 0.3 3.53 ± 1.2	3.23 ± 0.32 3.53 ± 0.83	1.17 ± 0.56 1 ± 0.33	1.03 ± 0.3 0.86 ± 0.3	4.3 ± 0.62 4.43 ± 0.9	2.16 ± 1.31 1.66 ± 0.86
[32]	Group(I) Group (II)	3.75 ± 0.62 3.91 ± 0.79	1.0 ± 0.60 1.50 ± 0.52	NR NR	NR NR	NR NR	NR NR	2.0 ± 0.42 1.66 ± 0.49	1.0 ± 0.00 1.3 ± 0.49	5.75 ± 0.86 5.58 ± 0.90	2.0 ± 0.60 2.83 ± 0.57
[33]	Group(I) Group (II)	3.17 ± 0.83 3.08 ± 0.79	0.83 ± 0.80 1.25 ± 0.83	NR NR	NR NR	NR NR	NR NR	1.00 ± 0.00 1.04 ± 0.14	0.79 ± 0.33 0.79 ± 0.54	4.16 ± 0.83 4.12 ± 0.80	1.70 ± 0.86 1.90 ± 0.94
[34]	Group(I) Group (II)	Mean (2.5) Mean (2.5)	Mean (0.54) Mean (1.5)	Mean (4) Mean (4.83)	Mean (1.33) Mean (3.5)	Mean (2.67) Mean (2.54)	Mean (3.42) Mean (2.83)	Mean (2.58) Mean (2.25)	Mean (2.08) Mean (2.17)	Mean (5.01) Mean (4.42)	Mean (2.92) Mean (3.83)
[17]	Group(I) Group (II)	NR NR	NR NR	NR NR	NR NR	NR 1.33 ± 0.50	NR 2.22 ± 0.67	NR NR	NR NR	4.89 ± 0.78 4.56 ± 0.73	2.44 ± 0.88 3.67 ± 0.71
[35]	Group(I) Group (II)	NR		NR		NR		1.400 ± 0.548 1.600 ± 0.548	(at 3 months)1.000 ± 0.000 (at 3 months)1.000 ± 0.000	4.110 ± 0.037 3.888 ± 0.425	(at 3 months)1.434 ± 0.408 (at 3 months)1.258 ± 0.375
[18]	Group(I) Group (II)	(0-6 M) 1.80 ± 0.92 (0-6 M) 1.50 ± 1.53	(0-18 M) 1.90 ± 1.88 (0-18 M) 1.50 ± 1.53	(0-6 M) 0.30 ± 0.48 (0-6 M) 0.20 ± 0.42	(0-18 M) 1.30 ± 0.48 (0-18 M) 0.30 ± 0.48	(0-6 M) 1.10 ± 1.10 (0-6 M) 0.80 ± 1.03	(0-18 M) 0.90 ± 1.37 (0-18 M) 0.80 ± 1.03	(0–6 M) 0.20 ± 0.63 (0-6 M) 0.30 ± 1.34	(0–18 M) 0.20 ± 0.63 (0-18 M) 0.30 ± 1.34	(0-6 M) 1.50 ± 1.35 (0-6 M) 1.00 ± 1.05	(0–18 M) 1.60 ± 1.26 (0-18 M) 0.90 ± 1.20
[36]	Group(I) Group (II)	Median = 4.00 (2.00–4.00) Median = 3.00 (2.00–4.00)	Median = 1.00 (0.00–2.000 Median = 1.00 (0.00–2.00)	NR NR	NR NR	Median = 3.00 (2.00–4.00) Median = 3.00 (2.00–4.00)	Median = 4.00 (3.00–5.00) Median = 3.00(3.00–4.00)	Median = 2.00 (1.00–2.00) Median = 1.00(1.00–2.00)	Median = 2.00 (2.00–3.00) Median = 2.00 (1.00–3.00)	Median = 5.00 (4.00–6.00) Median = 5.00 (4.00–6.00	Median = 3.00 (1.00–5.00) Median = 3.00 (1.00–5.00)

### Data extraction

The following data were extracted and recorded in duplicate by two independent reviewers (R.A and W.S): authors and year of publication, study design, randomization, the utilized surgical techniques, recession type, and location, number of surgical defects, follow-up period, percentage of root Coverage, the age and gender of the participants, and main authors’ conclusion.

### Risk of bias assessment

**Fig. 2:**
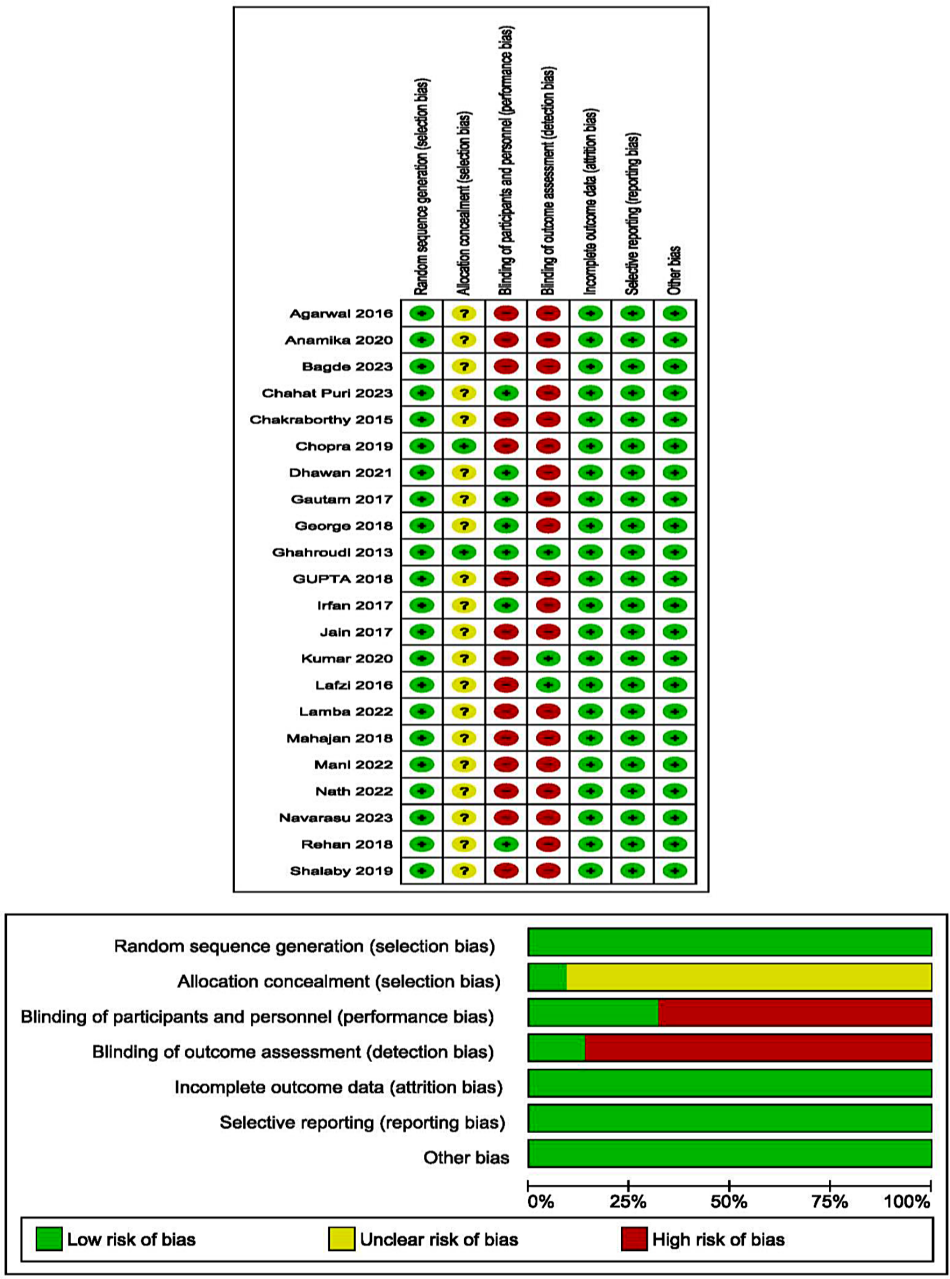
Risk of bias summary and graph

The risk of bias analysis for the included studies was performed using the Cochrane risk of bias tool (Revman 5.4, Version 5.4.1, Copenhagen, Denmark: The Nordic Cochrane Centre, The Cochrane Collaboration, 2020.) [37]. The included studies were assessed based on: Random sequence generation, allocation concealment, blinding of participants and personnel, blinding of outcome assessments, incomplete outcome data and selective reporting. These domains were graded as high, unclear or low risk based on individual assessments. Figure 2.

In our review, two independent investigators (R.A. and W.S.) applied the Cochrane Risk of Bias Tool to each included study. The studies were categorized into the following groups:

The studies were categorized into the following groups:

Low risk of bias: This category was assigned if all criteria were met or if one criterion was unclear or not met. This implies that the study demonstrated a high level of methodological rigor and minimized potential sources of bias.

Moderate risk of bias: Studies were categorized as having a moderate risk of bias if two criteria were unclear. This suggests that some aspects of the study design or conduct raised concerns about potential bias.

High risk of bias: A high risk of bias classification was given to studies where more than two criteria were not met, indicating a significant potential for bias in the study.

Any discrepancies or disagreements between the two investigators during the risk of bias assessment were addressed through discussion to ensure consistency and reliability in the assessment process.

### Statistical analysis

The meta-analysis of the included studies of this systematic review was conducted by the following software program (Revman5.4.1 (Review Manager Version 5.4.1(Revman5.4.1); The Cochrane Collaboration, Copenhagen, Denmark). The meta-analysis was conducted on the mean values of (RD, RW, WKG, and CAL) of the included studies at 6 months follow-up only. For the analysis of the continuous data as RD, RW, WKG, and CAL, the mean difference was measured with corresponding 95% confidence intervals (CI). When the result of the meta-analysis is of low heterogeneity (p ≥ 0.10, I² ≤ 50%), the fixed-effect model is used for the result comparison. The random-effect model is employed for comparing the result of the meta-analysis when the result heterogeneity is high (p < 0.10, I² > 50%).

The results of the meta-analysis were represented in the forest plot and the heterogeneity across studies in RD, RW, WKG, and CAL were correlated through subgroup analysis.

## Results

### Search outcomes

The search across the databases yielded a total of 264 potentially relevant articles. Both reviewers, R.A and W.S, screened 224 articles by reviewing the titles and abstracts. Out of these, 185 articles were excluded, leaving 39 articles for further investigation.

Both authors thoroughly reviewed the complete publications, and it was found that 17 of the articles did not meet the eligibility criteria. Consequently, 22 eligible articles were chosen. When examining the references in these 22 eligible articles, it was discovered that 11 publications had already been included in a previous meta-analysis [38], while 11 new publications were added to our selection. In total, we included 22 articles that met the eligibility criteria. These selected articles were published between 2013 and 2023, with the majority of them being randomized clinical studies. The selection process is illustrated in Fig. 1.

#### Primary outcomes

the primary outcomes included three measurements (RD, RW, and WKG) which were represented in the meta-analysis, and the forest plots figures.

1. **RD**: The meta-analysis of the reduction of RD included sixteen studies using the random effect model due to the detected heterogeneity found (I^2^ = 33%). We detected a statistically significant difference (p = 0.004) between the group of CAF & AM v/s CAF alone.

**Fig. 3:**
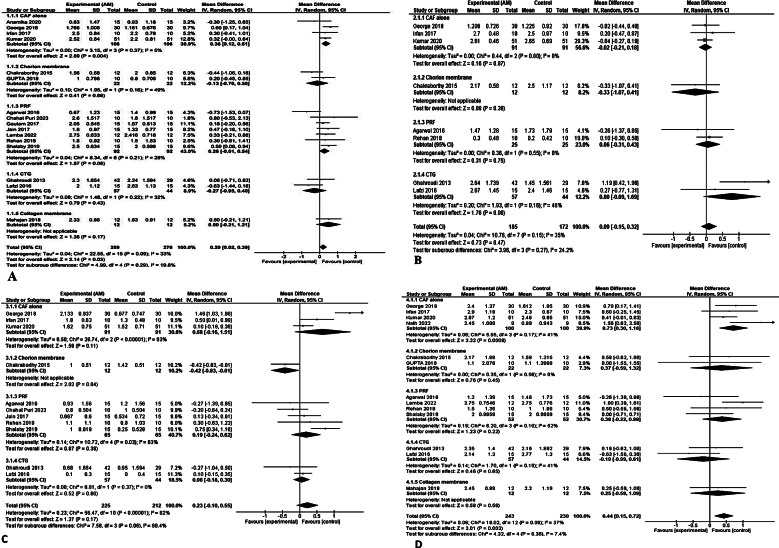
Forest plot of (**A**) RD reduction at 6 months; (**B**) RW reduction at 6 months; (**C**) WKG gain at 6 months; (**D**) CAL gain at 6 months

However, when comparing the (AM&CAF) group to the groups of CAF alone, platelet-rich fibrin (PRF), SCTG, Chorion membrane (CM), and Collagen membrane, we found that there were no significant differences (p = 0.29)” (Fig. 3A).

(2) **RW**: The random effect model was utilized in the RW meta-analysis of eight studies with heterogeneity found (I^2^ = 35%). There was no statistically significant difference between (AM&CAF) group when compared to (CAF alone, CM, PRF, and CTG) groups (p = 0.27) (Fig. 3B).
(3) **WKG**: Eleven studies were included in the meta-analysis of WKG gain with the use of a random effect model due to the heterogeneity found (I^2^ = 78%). The subgroup analysis comparing CAF with AM to CAF with CM revealed a notable increase in the WKG within the CM group (1.42 ± 0.51) in contrast to the AM group (1.00 ± 0.51). This disparity was statistically significant (p = 0.04).
(4) However, the overall comparison results between (AM&CAF) group, and (CAF alone, CM, PRF, and CTG) groups did not show any statistically significant difference (p = 0.31) (Fig. 3C).

### Secondary outcomes

One measurement was included in this meta-analysis (CAL) and represented by the forest plot.

#### Clinical attachment level (CAL)

Thirteen studies were included with the use of a random effect model due to the heterogeneity found (I^2^ = 37%). The subgroup analysis of CAF with AM v/s CAF alone showed a statistically significant difference (p = 0.0009) in favor of the control group (CAF alone). However, the overall comparison results between (AM & CAF), and (CAF alone, CM, PRF, SCTG, and Collagen membrane) groups did not show any statistically significant difference with (p = 0.36) (Fig. 3D).

Additionally, the funnel plots of the included parameters were expressed with the following conclusion: the funnel plots did not indicate any asymmetric distribution in all parameters, which showed no possible publication bias. All the studies were present inside the triangular area of the 95% CI region.

## Discussion

To the best of our knowledge, this systematic review and meta-analysis represent the most up-to-date investigation into the efficacy of Amniotic Membrane (AM) used in conjunction with Coronally Advanced Flap (CAF) when compared to the frequently reported treatment alternatives, namely CAF alone or in combination with other biomaterials (Platelet-Rich Fibrin (PRF), Collagen Membrane (CM), Subepithelial Connective Tissue Graft (SCTG), and collagen membrane). Previous systematic reviews have been hindered by a limited number of included studies [38, 39]. Our aim was to provide a comprehensive assessment of primary and secondary clinical outcomes.

The application of CAF in periodontal surgery has been associated with restricted tissue regeneration. This phenomenon is attributed to the development of a long junctional epithelium, characterized by the invasion of epithelial cells into the periodontal defect, resulting in tissue repair rather than true regeneration. To address this limitation, strategies aligned with the Guided Tissue Regeneration (GTR) principle have been adopted. These strategies involve integrating CAF with various biomaterials, including PRF, AM, collagen membrane, and acellular dermal matrix, all of which serve as barrier membranes with the goal of enhancing gingival coverage outcome [40].

Reconstructive surgery to treat periodontal defects includes numerous mucogingival esthetic surgeries which improve periodontal health by reconstruction of both lost hard and soft tissues. For the root coverage procedures, several techniques have emerged to impede further attachment loss and improve the esthetic outcomes. Recent techniques using AM have been introduced that permit root coverage with more promising results [41]. Recently, AM showed a good healing ability, exceptional biocompatibility, and easy availability at an affordable cost. So, it has been further suited for the treatment of different periodontal conditions such as furcation defects [42, 43].

However, the existing literature has not provided sufficient clinical evidence to conclusively determine the efficacy of AM when combined with CAF in treating GR. Hence, our systematic review was conducted to compare the effectiveness of AM with CAF in GR treatment among adult patients. We included twenty-two randomized clinical studies in this review, excluding studies with incomplete outcome data and inadequate follow-up periods from the meta-analysis [17, 21, 23, 24, 34, 35]. Consequently, we have compiled the findings of this systematic review to address clinical outcomes and provide recommendations regarding the utilization of AM with CAF in the treatment of GR, specifically Miller class I and II defects, in comparison to other established treatment modalities.

In our meta-analysis, we investigated the impact of AM on the reduction of RD after a 6-month interval from baseline. Sixteen studies were included in this analysis, revealing no statistically significant difference between the primary groups (p = 0.29). However, upon closer examination, we observed a significant reduction in RD in the subgroup that analyzed AM with CAF compared to CAF alone (p = 0.004). This effect can potentially be attributed to AM’s regenerative properties, which encompass growth factors, cytokines, extracellular matrix components, and bioactive compounds that may stimulate cell proliferation, migration, and differentiation [12]. Heterogeneity of RW seems to be linked to the different surgical techniques as the utilization of microsurgical protocol, the elevation of partial thickness flap, or the root surface bio-modification used by the application of ethylene di amine tetra acetic acid (EDTA) or tetracycline over the root surface [39].

Additionally, WKG and CAL gain were evaluated in eleven and thirteen studies, respectively, with no statistically significant differences detected between the overall groups (WKG: p = 0.31; CAL: p = 0.36). However, in studies comparing CAF with AM to CAF with CM, a statistically significant difference in WKG was found in favor of CM (p = 0.04).

For CAL, a statistically significant difference was found in the studies where AM was used along with CAF in comparison to AM alone. The gain in CAL may suggest a periodontal regeneration as well as a new epithelial attachment. However, the actual phenomenon behind the CAL gain is missing due to the lack of histological evidence in the included study of the current analysis [33].

Two studies [14, 31] examined the comparison between CAF combined with AM versus CAF combined with SCTG. In the test group (CAF + AM), RD and RW exhibited statistically significant differences in favor of the test group, indicating superior outcomes. This may be attributed to the enhanced potential of AM to stimulate creeping attachment. Conversely, in the CAF + SCTG group, a statistically significant difference was observed in CAL when compared to the CAF + AM group.

Three studies [25, 28, 30] examined the application of CAF combined with AM in comparison to CAF alone. The incorporation of AM into the CAF procedure did not result in statistically significant improvements across all measured parameters when compared to using CAF alone. This lack of improvement could potentially be attributed to the unfavorable placement of AM between an avascular surface (the tooth) and the flap, hindering the achievement of complete root coverage. Additionally, it’s worth noting that AM experiences some degree of shrinkage over time, leading to the creation of dead space between the root surface and surrounding tissues, which could potentially provide an environment for microorganisms and impede the healing process.

It can be concluded that AM shows comparable outcomes to other treatment modalities including CAF alone, CM, PRF, CTG, and collagen membrane as the AM graft is a reliable and viable method in GR treatment procedures and serves as a good alternative with uneventful healing and stable outcomes.

Our systematic review was conducted with a rigorous and up-to-date search strategy that aimed to encompass the most recent literature available up to our knowledge cutoff date. We employed an exhaustive search methodology, including multiple databases and grey literature sources, to ensure that we identified all relevant studies. While we aimed to include a comprehensive set of studies, we also maintained strict quality standards. Studies that did not meet our predefined quality criteria were excluded, ensuring that the included studies met high methodological standards. The inclusion of a larger number of studies in our meta-analysis can enhance the statistical power and precision of our findings, which can be especially important when investigating treatment effects in clinical research.

During our extensive database search, we stumbled upon a recently published meta-analysis [44]. Interestingly, this prior meta-analysis encompassed only eleven studies spanning from January 1, 2013, to December 31, 2020. However, in our current updated systematic review, our search extended until July 2023, culminating in the inclusion of an additional eleven recently published studies to complement the existing body of research. Consequently, the amalgamation of these studies resulted in a total of twenty-two published articles that met our stringent inclusion criteria for this review. The incorporation of these recent studies has significantly enriched our understanding and provided a comprehensive overview of all the available insights regarding the use of Amniotic Membrane (AM) as a contemporary biomaterial in conjunction with Coronally Advanced Flap (CAF) for the treatment of Gingival Recession (GR).

There are some reported limitations of the current meta-analysis, only twenty-two studies have been included so a larger number of RCTS is required to be conducted. Additionally, all analyses were done at six months follow-up so longer follow-up data should be planned by the researchers for more reliable results. Moreover, not all studies have reported the surgical methods utilized and the detailed methods of the randomization selection of their cases which may alter the outcomes. AM was fabricated by different companies so this might affect the membrane standardization with resultant different biological properties which might affect the outcomes.

In our meta-analysis, we observed a limited number of eligible studies that met our inclusion criteria. This scarcity of high-quality studies exploring the specific intervention may be due to the relatively recent emergence of this treatment approach or the strict inclusion criteria we applied to ensure methodological rigor. The meta-analyses with fewer than three studies can be subject to increased uncertainty. To address this limitation, we recommend the need for further research in the field to expand the available evidence base and to guide the clinicians in determining the most appropriate treatment when dealing with gingival recession.

## Conclusion

With the limitations of this meta-analysis due to short follow-up periods (6 months), the AM can recently be considered as a viable treatment option for Miller class I and II gingival recession defects with good outcomes comparable to other previously investigated modalities. Also, further well-designed clinical trials with long-term follow-up investigating the full potential of AM stem cell reservoir is still necessary to strengthen the fact that AM is truly a reservoir for periodontal tissue regeneration including GR treatment.

### Electronic supplementary material

Below is the link to the electronic supplementary material.

**Supplementary Material 1: Supplementary Fig. 1:** Funnel plot of RD reduction. **Fig. 2:** Funnel plot of RW reduction. **Fig. 3:** Funnel plot of WKG gain. **Fig. 4:** Funnel plot of CAL gain

## Data Availability

The data that support the findings of this study are available from the corresponding author upon reasonable request.
