# Supplementary material for: Efficacy of amniotic membrane with coronally advanced flap in the treatment of gingival recession: an updated systematic review and meta-analysis
Source: BMC Oral Health. 2024 Jan 25;24:133. doi: 10.1186/s12903-023-03825-y (PMC10811943; doi:10.1186/s12903-023-03825-y)
Supplement: Supplementary file 1 — Supplementary Material 1: Supplementary Fig. 1: Funnel plot of RD reduction. Fig. 2: Funnel plot of RW reduction. Fig. 3: Funnel plot of WKG gain. Fig. 4: Funnel plot of CAL gain [file 12903_2023_3825_MOESM1_ESM.docx]

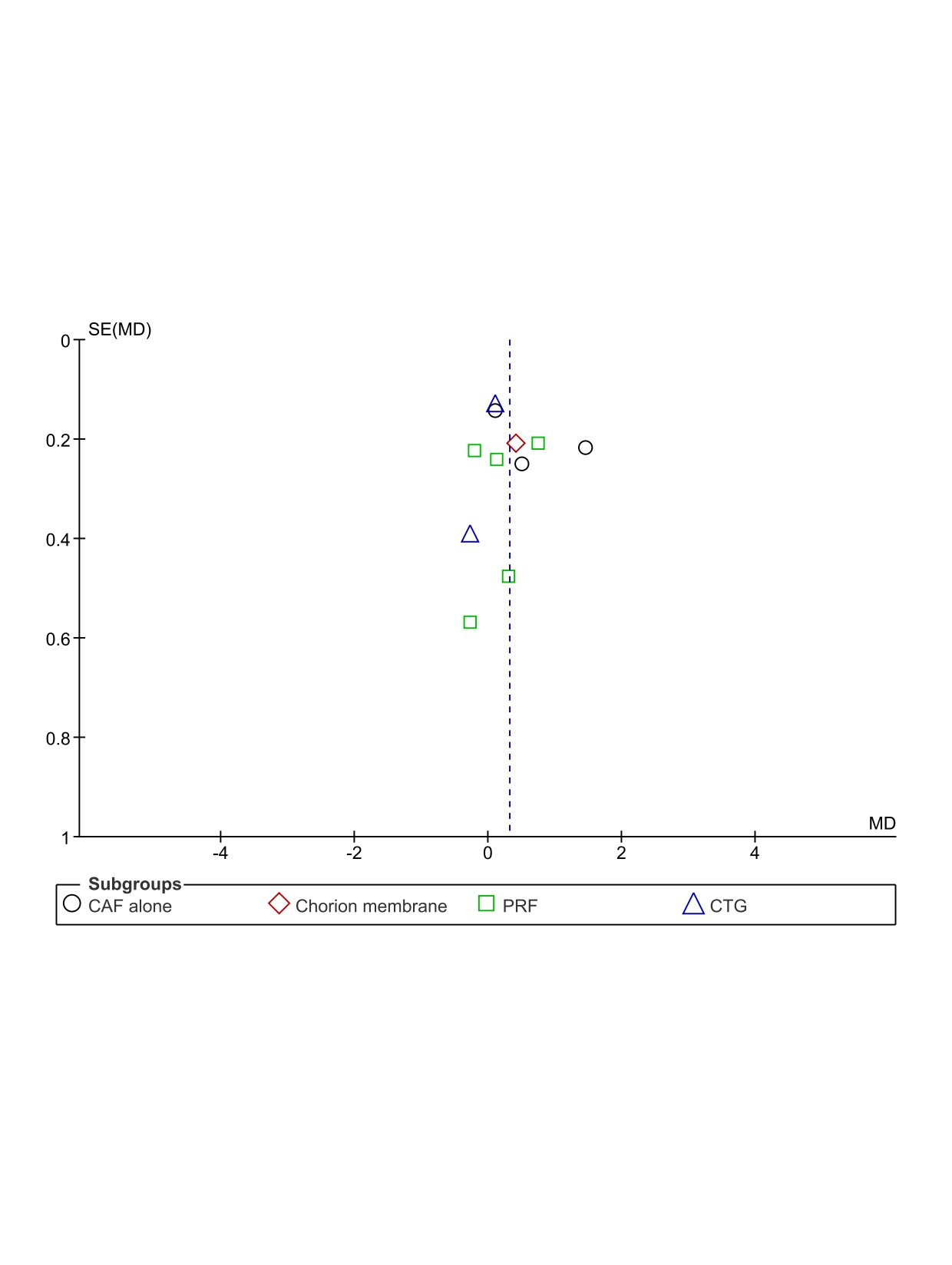


**Fig.1: Funnel plot of RD reduction**


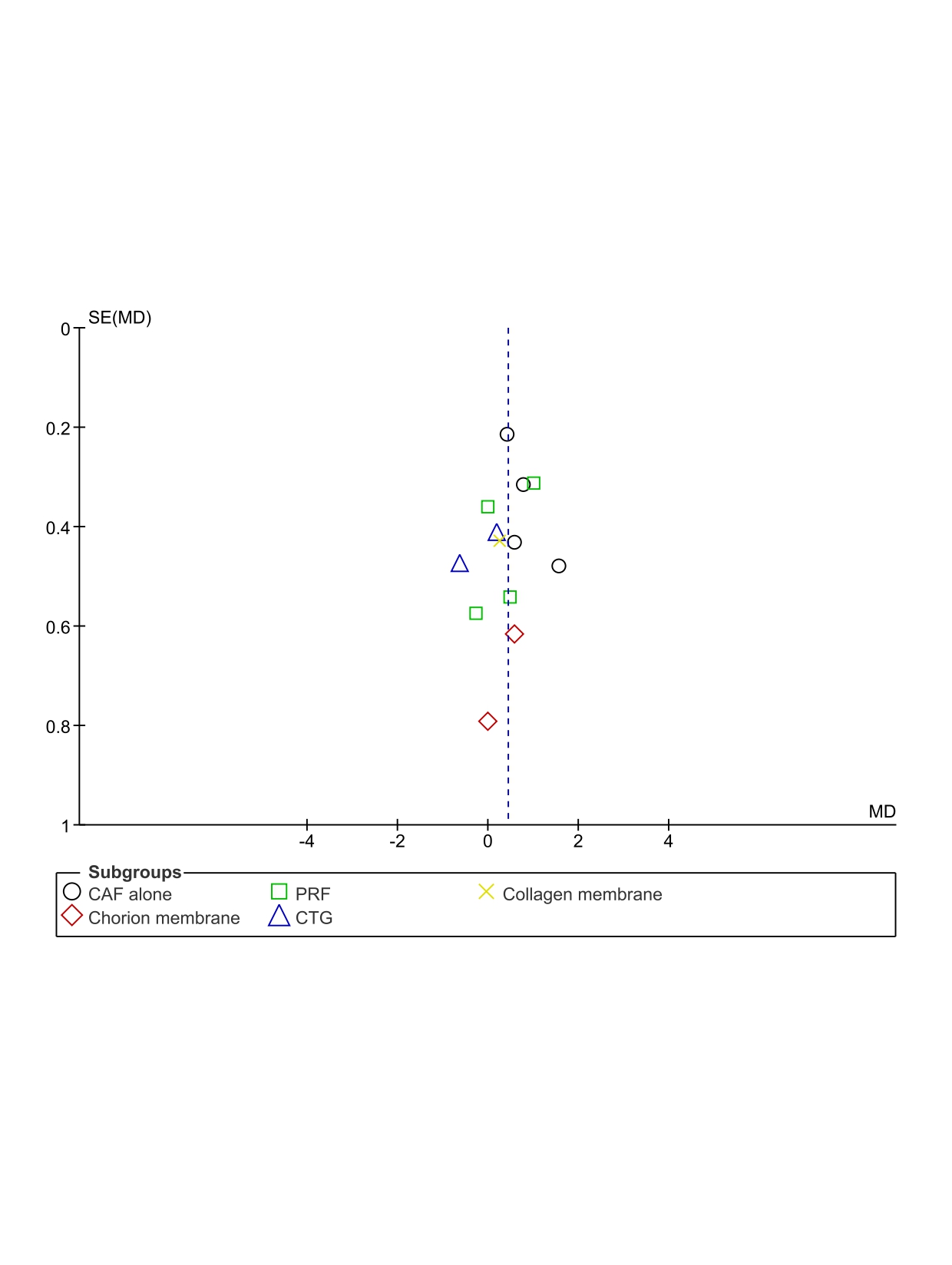


**Fig.2: Funnel plot of RW reduction**


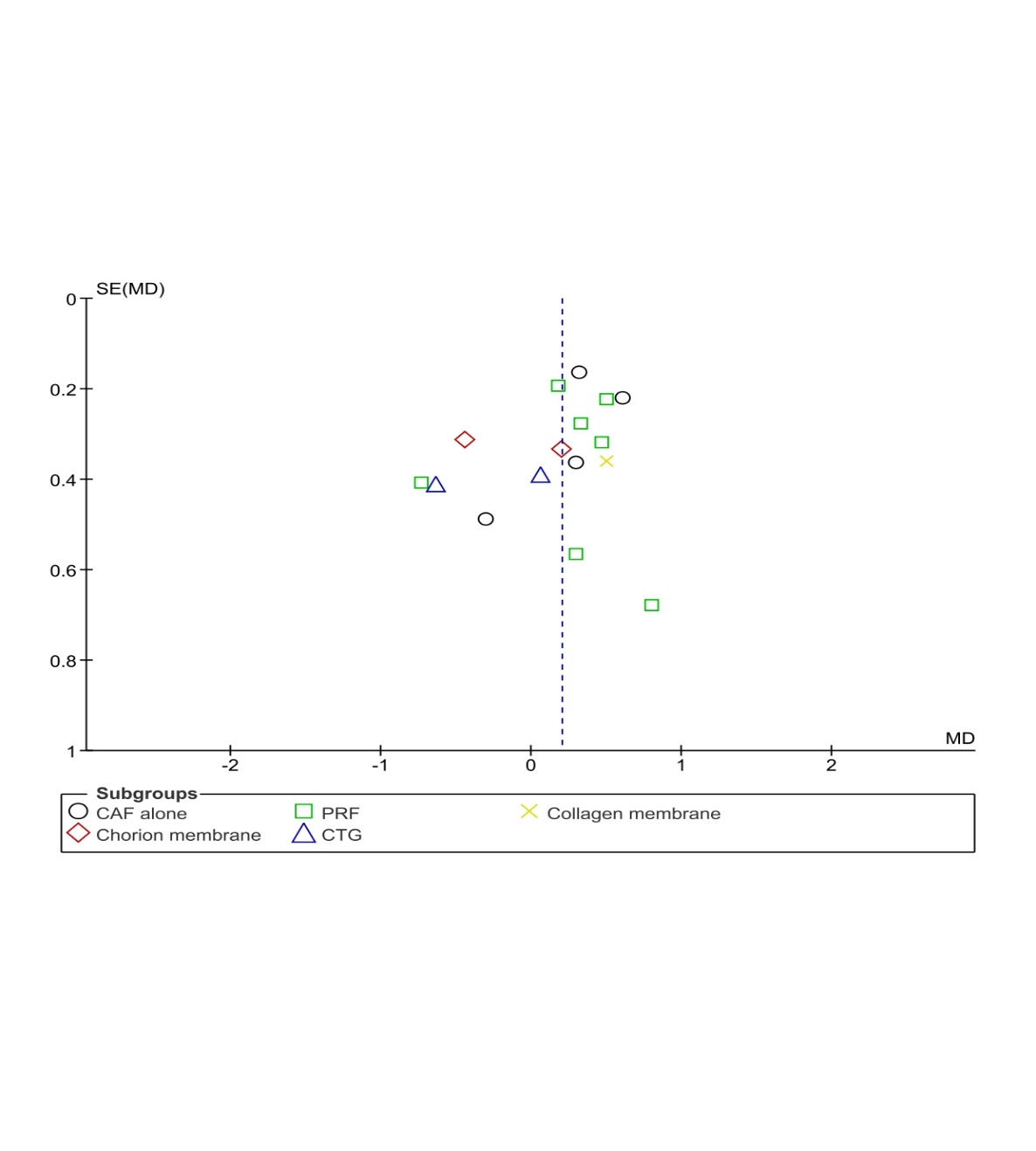


**Fig.3: Funnel plot of WKG gain**


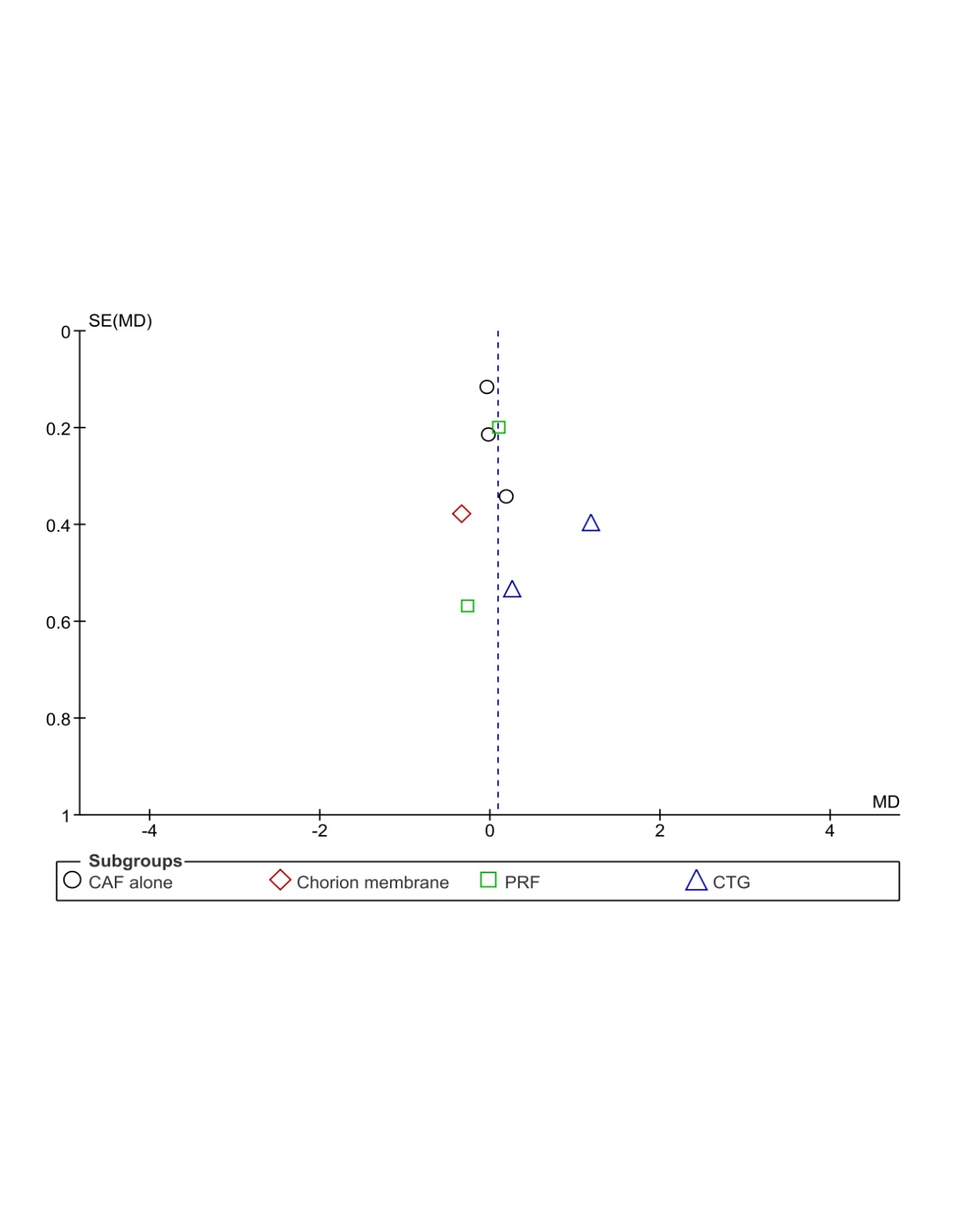


**Fig.4: Funnel plot of CAL gain**
